# Correlates of sedentary time in children: a multilevel modelling approach

**DOI:** 10.1186/1471-2458-14-890

**Published:** 2014-08-30

**Authors:** Thayse Natacha Gomes, Fernanda Karina dos Santos, Daniel Santos, Sara Pereira, Raquel Chaves, Peter Todd Katzmarzyk, José Maia

**Affiliations:** CIFI2D, Faculty of Sport, University of Porto, Rua Dr Plácido Costa, 91, Porto, 4200-450 Portugal; CAPES Foundation, Ministry of Education of Brazil, Brasília, DF Brazil; Federal University of Technology – Paraná (UTFPR), Campus Curitiba, Curitiba, Brazil; Pennington Biomedical Research Center, 6400 Perkins Road, Baton Rouge, LA 70808-4124 USA

**Keywords:** Sedentary behaviour, Children, School, Multilevel modelling

## Abstract

**Background:**

Sedentary behaviour (SB) has been implicated as a potential risk factor for chronic disease. Since children spend most of their awake time in schools, this study aimed to identify individual- and school-level correlates of sedentary time using a multilevel approach, and to determine if these correlates have a similar effect in normal weight (NW) and overweight/obese (O/O) children.

**Methods:**

Sample comprised 686 Portuguese children aged 9-10 years from 23 schools that took part in the ISCOLE project. Actigraph GT3X + accelerometers were used 24 hours/day for 7 days to assess sedentary time (daily minutes <100 counts/min); BMI was computed and WHO cut-points were used to classify subjects as NW or O/O. Sex, BMI, number of siblings, family income, computer use on school days, and sleep time on school days were used as individual-level correlates. At the school level, school size (number of students), percentage of students involved in sports or physical activity (PA) clubs, school promotion of active transportation, and students’ access to equipment outside school hours were used. All multilevel modelling analysis was done in SPSS, WINPEPI, and HLM.

**Results:**

School-level correlates explain ≈ 6.0% of the total variance in sedentary time. Results (β ± SE) showed that boys (-30.85 ± 5.23), children with more siblings (-8.56 ± 2.71) and those who sleep more (-17.78 ± 3.06) were less sedentary, while children with higher family income were more sedentary (4.32 ± 1.68). At the school level, no variable was significantly correlated with sedentary time. Among weight groups, variables related to sedentary time in NW were sex, sleep time and family income, while in O/O sex, number of siblings and sleep time were significant correlates. No school-level predictors were significantly associated in either of the weight groups.

**Conclusion:**

Notwithstanding the relevance of the school environment in the reduction of children’s sedentary time, individual and family characteristics played a more relevant role than the school context in this study.

## Background

Sedentariness is emerging as a potential risk factor for chronic disease [1–6]. For example, among adults, positive associations between sedentary behaviour (SB) such as sitting time and television viewing, and cardiovascular disease and adverse metabolic profiles have been reported [1–4]. In children, the link is also consistent between SB and increased prevalence of overweight/obesity [5], and an increase in metabolic risk factors [6]. Furthermore, systematic reviews have shown that screen time and overall sedentary time (objectively measured) track moderately during childhood and adolescence [7, 8], which means that reducing their sedentary time may be a way to induce health benefits into adulthood [9].

Understanding the correlates of sedentary time may aid in developing preventive strategies [10]. Sedentary time may be best represented by a construct that is different from physical activity (PA) [11, 12]; however, their determinants might be similar [11, 13]. Recently, it has been proposed that ecological approaches may provide a sound basis for a better understanding of sedentary time [14]. These approaches examine interactions between the subject and multiple levels of influence across intrapersonal (biological, psychological), interpersonal (social, cultural), organizational, physical environment (built, natural), and policy (laws, rules, regulations, codes) domains [10]. As such, factors that influence sedentary time in children could be different in home, neighbourhood and school settings, emphasising the necessity to understand the setting-specific multilevel factors that influence this complex behaviour.

Since children spend considerable time at school, this multifaceted environment could be an important venue for reducing their sedentary time. The school social and physical environments provide potential opportunities for children to avoid extended periods of sedentary time such as active transportation to and from school, large campus size or playground areas, sports equipment and sporting facilities, recess periods, lunch breaks, and physical education classes [15–19]. However, children spend most of their school time in sedentary activities [20]. The examination of school correlates of sedentary time among children, attempting to scrutinise the influence of factors coming from multiple levels, is not abundant in the literature [21, 22].

Given that students are influenced by shared and unique characteristics within and between schools, the correlates of sedentary time are ideally investigated using multilevel modelling [23]. Multilevel modelling analysis allows for the simultaneous examination of the effects of school- and individual-level predictors; accounts for the non-independence of observations within schools; does not treat subjects and school environment as unrelated, but they are seen as coming from a larger population; and examines both inter-individual and inter-school variation (as well as the contributions of school- and individual-level variables to these variations), allowing the investigation of individual and school contexts simultaneously [24–26].

The purposes of this study were to (1) estimate the between-school variability in sedentary time of Portuguese children, (2) identify individual- and school-level correlates of sedentary time, and also test cross-level interactions between BMI and school climate variables, and (3) determine if individual- and school-level correlates of sedentary time are similar among normal weight (NW) and overweight/obese (O/O) children.

## Methods

### Sample

A two-level random cluster sample of 777 5^th^ grade Portuguese children (419 girls, 358 boys) from 23 schools, aged 9-10 years old, was assessed. After exclusion criteria (non-valid accelerometer data), the final sample comprises 686 children (381 girls, 305 boys). The students were part of the International Study of Childhood Obesity, Lifestyle and the Environment (ISCOLE), a research project conducted at sites in 12 countries from all major world regions. In short, ISCOLE aims to determine the relationship between lifestyle behaviours and obesity in a multi-national study of children, and to investigate the influence of higher-order characteristics such as behavioural settings, and the physical, social and policy environments, on the observed relationships within and between countries [27].

After a first initial contact with a physical education teacher from each school, the project was presented to the physical education department. Following their approval, the project was then presented to the school principal as well as to the parental council; it was only after obtaining these consents that the project was implemented in each school. All 5^th^ grade children were invited to be part of the ISCOLE; however, only children aged between 9.5 and 10.5 years old were classified as “eligible” to be part at the project. From those “eligible” children, a sample of ≈ 30-40 children per school was randomly selected (50% for each gender). Non-response was negligible (response rate was 95.7%), and missing information was at random, since differences between subjects with missing information and those included in the present study were not statistically significant (data not shown).

Data were collected from September 2011 to January 2013. All assessments were done during a full week per school. The study protocol was approved by the University of Porto ethics committee, as well as by the schools’ directorate councils. Written informed consent was obtained from parents or legal guardians of all children. All data collection and management activities were performed and monitored under rigorous quality control procedures, implemented by the ISCOLE Coordinating Center, as described in detail by Katzmarzyk et al. [27].

### Anthropometry

Height and weight measures were obtained according to standardized ISCOLE procedures [27]. Each child was measured twice and, when necessary, a third measurement was taken if the difference between the previous two was outside the permissible range for each measure and its replica (0.5 cm for height and 0.5 kg for weight). The mean value of each measured variable was used for analysis.

Body mass index (BMI) was calculated using the standard formula [weight(kg)/height(m)^2^], and subjects were classified as normal weight, overweight, or obese according to the cut-off points from the World Health Organization (WHO) [28]. In the present paper, and to pursue our second aim, two BMI groups were formed: a NW group, and O/O group. Since the number of children classified as underweight was very low (8 cases), they were included in the NW group.

### Family data

Family information was obtained by a questionnaire completed by parents or legal guardians [see ISCOLE Demographic and Family Health Questionnaire in Katzmarzyk et al [27]]. The questionnaire collected information on basic demographics, ethnicity, family health and socioeconomic factors. For the present study, we only use information on family income [as an indicator of socioeconomic status (SES)] and number of siblings.

Subjects were classified into one of eight categories of annual family income, ranging from < €6000 to ≥ €42000, where category 1 represents lowest family income, and category 8 represents the highest. In the analysis strategy used in this paper, the family income socioeconomic variable was centered at category 4. Parents were also asked about family size, i.e., number of siblings.

### Sleep and sedentary time

Actigraph GT3X + accelerometers (ActiGraph, Pensacola, FL) were used to monitor sleep and sedentary time. Children wore the accelerometer at their waist on an elasticized belt, placed on the right mid-axillary line 24 hours/day, for 7 days, including 2 weekend days. To be eligible for this analysis, children had at least 4 days with a minimum of 10 hours of wear time per day; a total of 686 children fulfilled this condition.

Accelerometer data were first divided into awake time and nocturnal sleep time using an automated algorithm [29, 30]. After exclusion of the nocturnal sleep episode time, waking non-wear time was defined as any sequence of at least 20 consecutive minutes of zero activity counts [30].

Sedentariness is a multi-faceted characteristic that includes behaviour at work/school, at home, during transport, and in leisure-time including screen-time, motorized transportation, and sitting (to read, talk, do homework, or listen to music) [31]. In the present study, sedentary time was defined as minutes/day spent at less than 100 counts/min (using 1 minute epochs) as advocated by Treuth et al [32]. Further, information was also collected about children’s SB, by asking them about time spent in computer use during school days [ISCOLE Diet and Lifestyle Questionnaire, described in Katzmarzyk et al [27]].

### School environment

Information concerning the school environment (context and climate) was obtained via a questionnaire [ISCOLE School Environment Questionnaire presented in Katzmarzyk et al. [27]] which was completed by the physical education teacher or the school principal. The questionnaire includes items related to school facilities, healthy eating and PA policies, extracurricular activities, frequency of physical education and breaks (recess), promotion of active transportation, availability of healthy and unhealthy foods in the cafeteria and vending machines, number of days that students attend school during the academic year, and the amount of class time mandated for physical education. For the present study we considered primarily the (i) school context information regarding school size (number of students), and (ii) school climate which includes percentage of students participating in school sports or PA clubs, school promotion of active transportation (allowing children to bring their bicycles), and students’ access to sports equipment outside of school time. These variables were chosen firstly because there is evidence that they are correlated with PA and sedentariness in school children; and secondly, because of the multilevel data structure.

### Data analysis

Descriptive statistics, t and chi-square tests were computed in IBM SPSS version 20.0, and WinPepi version 11.26 [33]. Modelling the relationship between children’s sedentariness, their individual characteristics (level-1), and school environmental factors (level-2) was done in HLM 7.02 software within the framework of the multilevel approach using maximum likelihood estimation procedures [34].

A series of hierarchical nested models were fitted, and the Deviance statistic was used as a measurement of global fit. It is expected that as models increase in complexity by adding predictor variables, a significant decrease in Deviance is expected to occur, and the significance of the decrease is tested with a chi-square test [35]. In addition, the relevancy of predictors to explain SB was assessed with a pseudo-R^2^ statistic which is interpreted as a proportional reduction in variance for the parameter estimate that results from the use of one model as compared to a previous one [34]. Modelling was done in a “stepwise” fashion as generally advocated [see, for example, Hox [35], and Snijders and Bosker [36]]. Firstly, a null model (M_0_) was fitted to the data to compute the intraclass correlation coefficient to estimate the variance accounted for by school effects in sedentariness. Secondly, Model 1 (M_1_) was fitted to the data using only children predictors of sedentariness (gender, BMI, number of siblings, family income, computer use, and sleep time). BMI and sleep time were centered at the grand mean. Thirdly, Model 2 (M_2_) was fitted by adding all school predictors and cross-level interactions. This analysis was firstly done using the total sample (i.e., all subjects), and then repeated using the two sub-samples based on WHO cut-offs for BMI (NW and O/O).

## Results

Tables 1 and 2 show descriptive statistics (Mean ± SD and percentages) for level 1 and level 2 variables. Boys and girls had similar (p > 0.05) heights, weights, BMI, number of siblings, and mean sleep time. Also, no differences were found in overweight prevalence among genders (χ^2^ = 0.772, p = 0.380), but obesity had a higher frequency in boys (χ^2^ = 9.895, p = 0.002). Girls had higher sedentary time than boys (*t* = 6.085, p < 0.001).

**Table 1:** **Descriptive statistics for variables at the child level (level-1)**

	Child-level variables (mean ± SD)			
	**Boys (N = 305)**	**Girls (N = 381)**	***t***	***p-*** **value**
Height (cm)	143.46 ± 6.42	143.49 ± 7.06	0.060	0.952
Weight (kg)	40.52 ± 9.23	40.28 ± 9.23	-0.332	0.740
BMI (kg/m^2^)	19.54 ± 3.45	19.41 ± 3.36	-0.511	0.610
Number of siblings	0.97 ± 0.80	0.95 ± 0.83	-0.230	0.818
SED time	449.73 ± 73.07	482.21 ± 66.45	6.085	<0.001
Sleep time (hours/day)	8.14 ± 1.02	8.21 ± 0.96	0.964	0.335
	**BMI classification (%)**		**χ** ^**2**^	***p-*** **value**
Normal-weight	49.05%	58.0%	4.920	0.026
Overweight	15.1%	17.6%	0.772	0.380
Obese	35.4%	24.4%	9.895	0.002
	**Annual family income**			
Category 1	14.1%	22.7%		
Category 2	33.2%	29.3%		
Category 3	21.2%	16.7%		
Category 4	11.6%	9.3%		
Category 5	7.1%	7.0%		
Category 6	4.6%	6.0%		
Category 7	2.9%	3.7%		
Category 8	5.4%	5.3%		
	**Computer use on school days**			
Did not use	36.4%	46.2%		
<1 hour	23.3%	29.4%		
1 hour	21.0%	16.8%		
2 hours	12.8%	5.2%		
3 hours	3.3%	1.8%		
4 hours	1.6%	0.3%		
5 or more hours	1.6%	0.3%

**Table 2:** **Descriptive statistics for variables at the school level (level-2)**

School-level variables		
Number of students (mean ± SD)		782 ± 309
Children participation in sports or PA clubs		
	Not available	4.3%
	Less than 10%	4.3%
	10-24%	34.8%
	25-49%	13%
	≥50%	43.5%
Promoting active transportation (bike)		
	No	21.7%
	Yes	78.3%
Student’s access to equipment outside school hours		
	No	47.8%
	Yes	52.2%

More than 90% of the schools have children engaged in sports participation or PA clubs, more than 75% of them promote active transportation among their students, and about 50% of them allow the students to have access to sports equipment outside of school hours. The mean number of students per school is 782 ± 309, ranging from 239 to 1589.

Results of the null model, as well as for the other two models from the full sample, are presented in Table 3. Estimated variance at the school level suggests significant inter-individual differences across schools in sedentary time (χ^2^ = 67.32, p < 0.001). The estimated school-level effects from the intraclass correlation coefficient was 0.0609, meaning that ≈ 6.0% of the total variance in sedentary time among all children is explained by school effects, and 94% is explained by children’s distinct characteristics at their individual level. Also, the reliability estimate of 0.65 is an indicator of how well each school sample mean estimates the overall schools mean sedentary time parameter.

**Table 3:** **Results summary of hierarchical linear modelling for all sample: estimates, standard-errors, and p-values**

Parameters	Null model			Model 1			Model 2		
	***Estimates***	***Standard error***	***p-value***	***Estimates***	***Standard error***	***p-value***	***Estimates***	***Standard error***	***p-value***
Intercept	467.02	4.53	<0.001	484.46	5.67	<0.001	491.70	13.05	<0.001
Sex				-30.44	4.97	<0.001	-30.85	5.23	<0.001
BMI				-0.22	0.69	0.752	1.06	1.85	0.566
BMI X Participation in sports or PA clubs							-0.14	0.65	0.829
BMI X Promoting active transport							-1.75	1.74	0.316
BMI X Access to equipment outside school hours							0.74	1.67	0.656
Number of siblings				-8.50	2.67	0.002	-8.56	2.71	0.002
Family income				4.24	1.70	0.013	4.32	1.68	0.010
Computer using on school days				2.68	3.07	0.383	2.62	3.10	0.399
Sleep time				-17.90	2.96	<0.001	-17.78	3.06	<0.001
School size							-0.001	13.05	0.910
Participation in sports or PA clubs							-1.81	3.78	0.637
Promoting active transport							-2.33	8.53	0.787
*Variance components: random effects*									
School mean	309.50			202.89			190.05		
Children level effect	4765.57			3854.52			3852.97		
*Model summary*									
Deviance statistic	7781.24			5551.69			5550.73		
Number of estimated parameters	3			9			15		

Results from M_1_ related to individual-level predictors show that the sedentary time mean for a girl with a mean age of 10.5 years is 484 minutes · day^-1^. Boys, children with more siblings and those who sleep more are less sedentary, i.e. spend less time in sedentary activities (p < 0.05), but those with higher family income tend to be more sedentary (p = 0.013). No statistically significant associations were found for BMI and time spent using a computer on school days in mean sedentary time (*p* > 0.05). The reduction in the variance component at the children’s level allowed the estimation of the proportion (34.4%) of children’s characteristics explaining the inter-individual variance in sedentary time.

The final model, M_2_, investigated school effects as well as cross-level interactions. In this model, we assumed that the intercept parameter (sedentary time) varies at level 2. The mean sedentary time of a girl from a school where students are not involved in sports or PA clubs, and do not promote active transportation to school is 492 minutes · day^-1^. No significant associations were found for school size, percentage of students engaged in sports or PA clubs, or school promotion of active transportation. Similarly, cross-level interactions between BMI and school climate variables tested did not show any significant interaction.

Table 4 shows the results for the two weight groups (NW and O/O). Since BMI was used to classify subjects in weight groups, this variable was excluded in these analyses, as well the cross-level interactions between BMI and school climate variables. Among NW children, significant associations were found for sex, sleep time and family income, where boys and children who sleep more are less sedentary (p < 0.05); those with higher family income have higher sedentary time (p = 0.008). For O/O, being a boy, children with more siblings and those who sleep more have a significantly lower mean sedentary time (*p* < 0.001). Similar to the overall sample, no significant associations were found between sedentary time and school variables in NW and O/O groups.

**Table 4:** **Summary of results of final model two BMI groups (normal-weight and overweight/obese groups): estimates(standard-errors), and p-values**

Parameters	Normal-weight (N = 340)			Overweight/Obese (N = 272)		
***Regression coefficients: fixed effects***	***Estimates***	***Standard error***	***p-value***	***Estimates***	***Standard error***	***p-value***
Intercept	483.23	13.35	<0.001	514.03	19.96	<0.001
Sex	-32.93	8.45	<0.001	-29.12	7.33	<0.001
Number of siblings	-4.25	4.48	0.344	-10.25	5.18	0.049
Family income	6.29	2.34	0.008	2.59	2.58	0.317
Computer using on school days	1.63	3.52	0.643	5.44	4.44	0.222
Sleep time	-25.85	4.45	<0.001	-9.52	3.04	0.002
School size	-0.02	0.01	0.105	0.01	0.01	0.502
Participation in sports or PA clubs	-0.18	4.56	0.969	-6.20	5.75	0.294
Promoting active transport	7.67	8.85	0.397	-20.29	13.43	0.147
*Variance components: random effects*						
School mean	8.75			389.16		
Children level effect	4043.96			3492.64		
*Model summary*						
Deviance statistic	2919.96			2223.24		
Number of estimated parameters	11			11		

## Discussion

This study aimed to identify the magnitude of child- and school-level correlates of sedentary time and to determine if their importance was similar in NW and O/O children using a multilevel modelling approach.

At the child level, most of the variables included in the model were significantly linked to sedentary time. Sex differences in sedentary time are well documented [37], showing that girls spend more time in sedentary activities [11], which was confirmed in the present study. Van Stralen et al [38] studied the time devoted to sedentary activities at school in children aged 10-12 years from five European countries, and reported that girls spent a significant larger amount of school-time in sedentary activities (67%) than boys (63%, p < 0.0001), which can be related to differences in sex options for engagement in activities during recess time, with boys engaging more in competitive games while girls prefer socialising with friends [39]. Similarly, Verloigne et al [40] also found that girls spend more time in sedentary activities (511 minutes · day^-1^) than boys (478 minutes · day^-1^) taking into account the whole day, not only school time. Since in the present study children were monitored 24 hours · day^-1^, the sedentary time variable represents the entire day, not just sedentary time while at school. As such, in association with the explanation for the sex differences in sedentary time during school hours, it is also possible that these differences may be potentiated by dissimilarities in boys’ and girls’ leisure time activities. Since boys tend to devote more time in PA and/or in sports participation [41] during their leisure time, this behaviour may be relevant to decrease their sedentary time.

The influence of siblings on children’s sedentariness is not clear. It has also been reported, in a longitudinal study, that children with more siblings exhibit smaller increases in objectively measured sedentary time [42]. On the other hand, Verligne et al [43] investigated the effect of an intervention program on 10-12 year old Belgian children’s total sedentary time, and reported that those with one or more siblings were less likely to reduce sedentary time after the intervention program. Further, Tandon et al [44] reported that children watched TV/DVD’s with siblings more days per week, on average, than they did PA’s, reinforcing a potentially positive influence of the sibling for SB. On the other hand, it was suggested that the presence of more children at home (i.e., more siblings) is highly related with more moderate-to-vigorous PA overall and at home, and more sedentary time at home but less screen time [45]. We found a negative association between number of siblings and sedentary time in children, implying that the more siblings children have, the less sedentary they are. Since at this age there is a high peer influence in children behaviour [41], it is possible that those with less sedentary siblings tend to also become less sedentary.

Sleep time was negatively associated with sedentary time, indicating that children that slept more spent less time in sedentary activities. Several studies have shown that SB may interfere with sleep [46–48], but the results are not conclusive. For example, Belgium students who spent more time in sedentary activities, such as watching TV, playing video games, and using the internet went to bed later, spending less time in bed on weekdays [47]. However, in Taiwanese adolescents [49] no association was found between the time they spent watching TV or using a computer and getting sufficient sleep.

A positive association was found between family income and sedentary time, although the results from other studies have not always been clear about the magnitude and direction of this association [11]. For example, Olds et al [50] studied the socio-demographic correlates of SB in children aged 9-16 years, and found that children from higher SES reported greater engagement in non-screen sedentary time (such as sitting or lying down), but those from lower SES spent more time in screen-based sedentary time (watching TV, playing videogames, using computer), and no significant difference across income bands was found for total sedentary time (sum of non-screen sedentary time and screen sedentary time). Similarly, Foley et al [51] reported that 10-18 years old adolescents from areas of lower deprivation (i.e., higher SES) tended to accumulate more total sedentary time, which was determined by the concomitant use of an accelerometer and a recall diary. Furthermore, Klitsie et al [52], also using an objective and subjective method to access sedentariness, reported that 9-10 year old children with higher SES spent more time in non-screen SB; however, those from low SES and those from high SES both had higher sedentary time than those of medium SES. Using an objective method to measure sedentary time, namely accelerometers, Steele et al [53] did not find any difference in sedentary time according to SES, while Atkin et al [42] reported an increase in sedentary time, after a one-year period, among children from higher SES. Our findings add to this body of evidence, and suggest that Portuguese children with higher family income have greater sedentary time than those with low family income.

There is some prior evidence that children with a higher BMI are more sedentary, spending more time watching TV [54, 55]. However, in the present study no significant association was found between sedentary time and BMI. Further, the interaction between BMI and school climate variable did not reveal a mediation effect of school characteristics on the role of BMI on sedentary time. However, TV watching was not specifically measured in the present study, and the relationship with BMI may differ across different sedentary behaviours.

Schools offer extracurricular activities and policies that could potentially reduce sedentary time among students [15–19]. In this study, only 6.0% of the total variance in sedentary time was explained by school-level variables. It is known that schools with a larger campus size or playground areas provide more opportunities for their students to engage in PA during recess time, potentially decreasing their sedentary time [15, 17, 19]. In addition, athletic facilities such as school sports or PA clubs appear to be good opportunities to decrease sedentary time and increase PA in youth [56]. Moreover, active commuting to school is associated with higher PA levels among youth [57, 58], and children who drive to/from school are less likely to achieve recommended levels of daily PA [59]. However, despite the suggestion that school context has the potential to reduce children’s sedentary time, in the present study we did not find such an association. Our study was potentially under-powered to identify school level effects, given the sample size of only 23 schools (versus a sample size of 686 children for individual-level correlates). Further, there was limited variance in some of the school-level variables measured in this study (i.e. more than 90% of the schools have children engaged in sports participation or PA clubs). Thus, a study with a larger sample size of school, and with greater variability among schools in the environmental variables, may be better suited to detect school-level correlates.

When the analyses was stratified by body weight status, sex and sleep time were related to sedentary time in both NW and O/O groups; family income was only related to sedentary time in the NW group, while number of siblings was related to sedentary time in O/O; further, no school-level predictor was significantly associated with sedentary time in either group. Differences in individual-level sedentariness correlates among weight groups suggests that attention should be paid to weight status when implementing strategies to decrease sedentary time in children, such that the chosen activities should be easily and playfully performed by both NW and O/O children; additionally, body weight should not be a barrier to those children with higher weight.

This study has several limitations and strengths. Firstly, as we did not study distinct SB’s (screen time, reading, listening to music, transportation to/from school, etc.), rather we focused on objectively determined overall sedentary time. Thus, it was not always possible to compare our results with previous studies that did not assess sedentary time objectively using accelerometry [11, 60]. Secondly, the present sample comes from only one Portuguese region and its results do not necessarily generalize to all children. However, a comparison of the present sample characteristics with information available from the Portuguese population of the same age and gender was done. For example, in data not shown here, no differences were found in the prevalence of overweight/obesity [61], in the percentage of children attaining sufficient levels of PA [62], and SES distribution [63]. Thirdly, despite the evidence that moderate-to-vigorous PA attenuates the association between SB and health risk [64], we did not include this information as a covariate. Notwithstanding these limitations, the study has several important strengths: (1) the use of an objective method to estimate sedentary time; (2) the use of the accelerometer for 7 days; (3) inclusion of objective information regarding sleep time; (4) using standard methods and highly reliable data, and (5) the use of multilevel modelling to capture the complexity of nested information available at the child and school levels.

## Conclusions

In summary, this study investigated the role of individual- and school-level variables with children’s sedentary time within the multilevel modelling framework. School context explained 6.0% of the total variance in children’s sedentary time. At the individual level, sex, number of siblings, family income and sleep time explained 34.4% of the 94% variance fraction of the individual level. No significant association was found between sedentary time and BMI, as well as between sedentary time and school-level correlates. Notwithstanding the relevancy of school diversified environments to reduce sedentary time in children, enhancing their opportunities for being less sedentary in their awake time, requires further analysis with a more diversified list of markers than those explored in the present study. Furthermore, differences in sedentary time correlates among NW and O/O children suggest that different strategies may be needed to reduce sedentary time in these two groups. Moreover, given the association between sedentary time and health risks, future studies should be conducted using direct measures of total sedentary time, distinguishing different types of SB and examining different patterns in which sedentary time is accumulated. Furthermore, the use of an inclinometer, in association with the accelerometer, could be useful to provide information regarding postural changes. In addition, since sedentariness and PA are two distinct phenotypes, and being physically active does not imply being less sedentary, future studies should also investigate the relationship between these two variables on health risk factors, independently and in association.

## Abbreviations

SB: Sedentary behaviour
PA: Physical activity
NW: Normal weight
O/O: Overweight/obese
ISCOLE: International study of childhood obesity, lifestyle and the environment
BMI: Body mass index
WHO: World health organization
SES: Socioeconomic status.

## References

[1] Katzmarzyk PT, Church TS, Craig CL, Bouchard C (2009). Sitting time and mortality from all causes, cardiovascular disease, and cancer. Med Sci Sports Exerc.

[2] Dunstan DW, Barr EL, Healy GN, Salmon J, Shaw JE, Balkau B, Magliano DJ, Cameron AJ, Zimmet PZ, Owen N (2010). Television viewing time and mortality: the Australian Diabetes, Obesity and Lifestyle Study (AusDiab). Circulation.

[3] Chau JY, Grunseit AC, Chey T, Stamatakis E, Brown WJ, Matthews CE, Bauman AE, van der Ploeg HP (2013). Daily sitting time and all-cause mortality: a meta-analysis. PLoS One.

[4] Thorp AA, Healy GN, Owen N, Salmon J, Ball K, Shaw JE, Zimmet PZ, Dunstan DW (2010). Deleterious associations of sitting time and television viewing time with cardiometabolic risk biomarkers: Australian Diabetes, Obesity and Lifestyle (AusDiab) study 2004-2005. Diabetes Care.

[5] Vicente-Rodriguez G, Rey-Lopez JP, Martin-Matillas M, Moreno LA, Warnberg J, Redondo C, Tercedor P, Delgado M, Marcos A, Castillo M, Bueno M (2008). Television watching, videogames, and excess of body fat in Spanish adolescents: the AVENA study. Nutrition.

[6] Steele RM, Brage S, Corder K, Wareham NJ, Ekelund U (2008). Physical activity, cardiorespiratory fitness, and the metabolic syndrome in youth. J Appl Physiol.

[7] Jones RA, Hinkley T, Okely AD, Salmon J (2013). Tracking physical activity and sedentary behavior in childhood: a systematic review. Am J Prev Med.

[8] Biddle SJ, Pearson N, Ross GM, Braithwaite R (2010). Tracking of sedentary behaviours of young people: a systematic review. Prev Med.

[9] Kohl HW, Hobbs KE (1998). Development of physical activity behaviors among children and adolescents. Pediatrics.

[10] Owen N, Sugiyama T, Eakin EE, Gardiner PA, Tremblay MS, Sallis JF (2011). Adults’ sedentary behavior determinants and interventions. Am J Prev Med.

[11] Pate RR, Mitchell JA, Byun W, Dowda M (2011). Sedentary behaviour in youth. Br J Sports Med.

[12] Katzmarzyk PT (2010). Physical activity, sedentary behavior, and health: paradigm paralysis or paradigm shift?. Diabetes.

[13] Uijtdewilligen L, Nauta J, Singh AS, van Mechelen W, Twisk JW, van der Horst K, Chinapaw MJ (2011). Determinants of physical activity and sedentary behaviour in young people: a review and quality synthesis of prospective studies. Br J Sports Med.

[14] Sallis J, Owen N, Glanz K, Lewis F, Rimer B (2001). Ecological models of health behaviour. Health behaviour and health education: theory, research and practice.

[15] Ridgers ND, Stratton G, Fairclough SJ, Twisk JW (2007). Long-term effects of a playground markings and physical structures on children's recess physical activity levels. Prev Med.

[16] Verstraete SJ, Cardon GM, De Clercq DL, De Bourdeaudhuij IM (2006). Increasing children's physical activity levels during recess periods in elementary schools: the effects of providing game equipment. Eur J Public Health.

[17] Cradock AL, Melly SJ, Allen JG, Morris JS, Gortmaker SL (2007). Characteristics of school campuses and physical activity among youth. Am J Prev Med.

[18] Wechsler H, Devereaux RS, Davis M, Collins J (2000). Using the school environment to promote physical activity and healthy eating. Prev Med.

[19] Sallis JF, Conway TL, Prochaska JJ, McKenzie TL, Marshall SJ, Brown M (2001). The association of school environments with youth physical activity. Am J Public Health.

[20] Fairclough SJ, Butcher ZH, Stratton G (2008). Primary school children's health-enhancing physical activity patterns: the school as a significant environment?. Education 3-13.

[21] Ridgers ND, Timperio A, Crawford D, Salmon J (2013). What factors are associated with adolescents’ school break time physical activity and sedentary time?. PLoS One.

[22] Mantjes JA, Jones AP, Corder K, Jones NR, Harrison F, Griffin SJ, van Sluijs EM (2012). School related factors and 1 yr change in physical activity amongst 9-11 year old English schoolchildren. Int J Behav Nutr Phys Act.

[23] Haug E, Torsheim T, Samdal O (2008). Physical environmental characteristics and individual interests as correlates of physical activity in Norwegian secondary schools: the health behaviour in school-aged children study. Int J Behav Nutr Phys Act.

[24] Diez-Roux AV (2000). Multilevel analysis in public health research. Annu Rev Public Health.

[25] Duncan C, Jones K, Moon G (1998). Context, composition and heterogeneity: using multilevel models in health research. Soc Sci Med.

[26] Snikders TAB, Bosker RJ (1994). Modeled Variance in Two-Level Models. Socl Meth Res.

[27] Katzmarzyk PT, Barreira TV, Broyles ST, Champagne CM, Chaput JP, Fogelholm M, Hu G, Johnson WD, Kuriyan R, Kurpad A, Lambert EV, Maher C, Maia J, Matsudo V, Olds T, Onywera V, Sarmiento OL, Standage M, Tremblay MS, Tudor-Locke C, Zhao P, Church TS (2013). The International Study of Childhood Obesity, Lifestyle and the Environment (ISCOLE): design and methods. BMC Public Health.

[28] de Onis M, Onyango AW, Borghi E, Siyam A, Nishida C, Siekmann J (2007). Development of a WHO growth reference for school-aged children and adolescents. Bull World Health Organ.

[29] Tudor-Locke C, Barreira TV, Schuna JM, Mire EF, Katzmarzyk PT (2014). Fully automated waist-worn accelerometer algorithm for detecting children's sleep-period time separate from 24-h physical activity or sedentary behaviors. Appl Physiol Nutr Metab.

[30] Barreira TV, Schuna JM Jr, Mire EF, Katzmarzyk PT, Chaput J-P, Leduc G, Tudor-Locke C: **Identifying children's nocturnal sleep using 24-hour waist accelerometry.** *Med Sci Sports Exerc* In press 10.1249/MSS.0000000000000486 25202840

[31] Biddle S, Cavil N, Ekelund U, Gorely T, Griffiths M, Jago R, Oppert J-M, Raats M, Salmon J, Stratton G, Vicente-Rodríguez G, Butland B, Prosser L, Richardson D (2010). Sedentary behaviour and obesity: Review of the current scientific evidence.

[32] Treuth MS, Schmitz K, Catellier DJ, McMurray RG, Murray DM, Almeida MJ, Going S, Norman JE, Pate R (2004). Defining accelerometer thresholds for activity intensities in adolescent girls. Med Sci Sports Exerc.

[33] Abramson J (2011). WINPEPI updated: computer programs for epidemiologists, and their teaching potential. Epidemiol Perspect Innov.

[34] Raudenbush SW, Bryk AS, Cheong YF, Congdon RT (2011). HLM 7: Hierarchical Linear and Nonlinear Modeling.

[35] Hox JJ (2010). Miltilevel analysis: Techniques and applications.

[36] Snijders TAB, Bosker R (2012). Multilevel Analysis: An Introduction to Basic and Advanced Multilevel Modeling.

[37] Van Der Horst K, Paw MJ, Twisk JW, Van Mechelen W (2007). A brief review on correlates of physical activity and sedentariness in youth. Med Sci Sports Exerc.

[38] van Stralen MM, Yildirim M, Wulp A, te Velde SJ, Verloigne M, Doessegger A, Androutsos O, Kovacs E, Brug J, Chinapaw MJ (2014). Measured sedentary time and physical activity during the school day of European 10- to 12-year-old children: the ENERGY project. J Sci Med Sport.

[39] Blatchford P, Baines E, Pellegrini A (2003). The social context of school playground games: Sex and ethnic differences, and changes over time after entry to junior school. Br J Dev Psychol.

[40] Verloigne M, Van Lippevelde W, Maes L, Yildirim M, Chinapaw M, Manios Y, Androutsos O, Kovacs E, Bringolf-Isler B, Brug J, De Bourdeaudhuij I (2012). Levels of physical activity and sedentary time among 10- to 12-year-old boys and girls across 5 European countries using accelerometers: an observational study within the ENERGY-project. Int J Behav Nutr Phys Act.

[41] Seabra AF, Mendonça DM, Thomis MA, Anjos LA, Maia JA (2008). Biological and socio-cultural determinants of physical activity in adolescents. Cad Saude Publica.

[42] Atkin AJ, Corder K, Ekelund U, Wijndaele K, Griffin SJ, van Sluijs EM (2013). Determinants of change in children's sedentary time. PLoS One.

[43] Verloigne M, Bere E, Van Lippevelde W, Maes L, Lien N, Vik FN, Brug J, Cardon G, De Bourdeaudhuij I (2012). The effect of the UP4FUN pilot intervention on objectively measured sedentary time and physical activity in 10-12 year old children in Belgium: the ENERGY-project. BMC Public Health.

[44] Tandon PS, Zhou C, Sallis JF, Cain KL, Frank LD, Saelens BE (2012). Home environment relationships with children's physical activity, sedentary time, and screen time by socioeconomic status. Int J Behav Nutr Phys Act.

[45] Tandon P, Grow HM, Couch S, Glanz K, Sallis JF, Frank LD, Saelens BE (2014). Physical and social home environment in relation to children's overall and home-based physical activity and sedentary time. Prev Med.

[46] Johnson JG, Cohen P, Kasen S, First MB, Brook JS (2004). Association between television viewing and sleep problems during adolescence and early adulthood. Arch Pediatr Adolesc Med.

[47] Van den Bulck J (2004). Television viewing, computer game playing, and Internet use and self-reported time to bed and time out of bed in secondary-school children. Sleep.

[48] Dworak M, Schierl T, Bruns T, Struder HK (2007). Impact of singular excessive computer game and television exposure on sleep patterns and memory performance of school-aged children. Pediatrics.

[49] Chen MY, Wang EK, Jeng YJ (2006). Adequate sleep among adolescents is positively associated with health status and health-related behaviors. BMC Public Health.

[50] Olds TS, Maher CA, Ridley K, Kittel DM (2010). Descriptive epidemiology of screen and non-screen sedentary time in adolescents: a cross sectional study. Int J Behav Nutr Phys Act.

[51] Foley LS, Maddison R, Jiang Y, Olds T, Ridley K (2011). It's not just the television: survey analysis of sedentary behaviour in New Zealand young people. Int J Behav Nutr Phys Act.

[52] Klitsie T, Corder K, Visscher TL, Atkin AJ, Jones AP, van Sluijs EM (2013). Children's sedentary behaviour: descriptive epidemiology and associations with objectively-measured sedentary time. BMC Public Health.

[53] Steele RM, van Sluijs EM, Sharp SJ, Landsbaugh JR, Ekelund U, Griffin SJ (2010). An investigation of patterns of children's sedentary and vigorous physical activity throughout the week. Int J Behav Nutr Phys Act.

[54] Crespo CJ, Smit E, Troiano RP, Bartlett SJ, Macera CA, Andersen RE (2001). Television watching, energy intake, and obesity in US children: results from the third National Health and Nutrition Examination Survey, 1988-1994. Arch Pediatr Adolesc Med.

[55] Gable S, Chang Y, Krull JL (2007). Television watching and frequency of family meals are predictive of overweight onset and persistence in a national sample of school-aged children. J Am Diet Assoc.

[56] Perkins DF, Jocobs JE, Barber BL, Eccles JS (2004). Childhood and adolescent sports participation as predicators of participation in sports and physical fitness activities during young adulthood. Youth Soc.

[57] Faulkner GEJ, Buliung RN, Flora PK, Fusco C (2009). Active school transport, physical activity levels and body weight of children and youth: A systematic review. Prev Med.

[58] Pabayo R, Maximova K, Spence JC, Vander Ploeg K, Wu B, Veugelers PJ (2012). The importance of Active Transportation to and from school for daily physical activity among children. Prev Med.

[59] Trapp G, Giles-Corti B, Christian H, Timperio AF, McCormack GR, Bulsara M, Villanueva K (2013). Driving down daily step counts: the impact of being driven to school on physical activity and sedentary behavior. Pediatr Exerc Sci.

[60] Pate RR, O'Neill JR, Lobelo F (2008). The evolving definition of “sedentary”. Exerc Sport Sci Rev.

[61] Sardinha LB, Santos R, Vale S, Silva AM, Ferreira JP, Raimundo AM, Moreira H, Baptista F, Mota J (2011). Prevalence of overweight and obesity among Portuguese youth: a study in a representative sample of 10-18-year-old children and adolescents. Int J Pediatr Obes.

[62] Baptista F, Santos DA, Silva AM, Mota J, Santos R, Vale S, Ferreira JP, Raimundo AM, Moreira H, Sardinha LB (2012). Prevalence of the Portuguese population attaining sufficient physical activity. Med Sci Sports Exerc.

[63] Fundação Francisco Manuel dos Santos (2013). PORDATA.

[64] Steele RM, van Sluijs EM, Cassidy A, Griffin SJ, Ekelund U (2009). Targeting sedentary time or moderate- and vigorous-intensity activity: independent relations with adiposity in a population-based sample of 10-y-old British children. Am J Clin Nutr.

[CR65] The pre-publication history for this paper can be accessed here:http://www.biomedcentral.com/1471-2458/14/890/prepub

